# CaZAT5 delays the flowering time in tomato and affects pollen viability and anther dehiscence

**DOI:** 10.1371/journal.pgen.1012016

**Published:** 2026-01-06

**Authors:** Jiachang Xiao, Min Yang, Junqi Yang, Wen Tang, Xueping Song, Yi Tang, Bo Sun, Yangxia Zheng, Zhi Huang, Huanxiu Li

**Affiliations:** College of Horticulture, Sichuan Agricultural University, Chengdu, China; Tsinghua University, CHINA

## Abstract

Male sterility (MS) plays a crucial role in plant reproduction and hybrid breeding as it is associated with pollen viability and release. However, the regulatory mechanisms governing anther dehiscence in peppers remain poorly characterized. Thus, this study identified the pepper C_2_H_2_ family transcription factor *CaZAT5* and characterized its function. The results indicated that *CaZAT5* represses transcriptional activity and is predominantly expressed during pepper flower development. Silencing *CaZAT5* in pepper led to early flowering, whereas its overexpression (OE) in tomato delayed flowering. Moreover, *Ca*ZAT5 negatively regulated vegetative growth by suppressing *CaSOC1* expression, thereby affecting pollen morphology and viability. Histological analyses revealed that the anthers of *CaZAT5-*OE plants exhibited abnormal mitosis, resulting in both enlarged and shrunken pollen grains. Additionally, *CaZAT5* overexpression inhibited anther dehiscence during pollen maturation, affecting pollen release. The consequent reduction in pollen viability and inhibited anther dehiscence decreased fruit set and yield in the plants. Transcriptome (RNA-seq) analysis revealed that *CaZAT5* overexpression suppressed the expression of genes involved in cell wall loosening, degradation, and secondary wall thickening in the anthers. DAP-seq, Y1H, Dual-LUC, and EMSA identified potential *Ca*ZAT5-regulated genes involved in anther dehiscence, including cell wall degradation genes (*CaPG* and *CaBG4*) and the expansin gene *CaExpA13*. Collectively, these findings suggest that *CaZAT5* modulates flowering time, pollen development, and anther dehiscence by regulating the expression of genes related to flowering and cell wall loosening and degradation. These findings contribute to a more comprehensive understanding of the potential role of *CaZAT5* in regulating flowering time and male fertility.

## Author summary

Functional male sterility is a reproductive phenomenon in which plants produce pollen, but sterility arises due to defective anther dehiscence and subsequent failure of pollen grains. This type of sterility enables fertility restoration through artificial pollination or hybridization. *Ca*ZAT5 encodes a C2H2-type transcription factor that plays broad roles in the transcriptional regulation of flowering induction, floral organ development, as well as pollen and pistil maturation. This study demonstrates that *Ca*ZAT5 regulates both flowering time and male fertility. *Ca*ZAT5 delays flowering by suppressing *CaSOC1* expression. Remarkably, *CaZAT5* overexpression in tomato resulted in two distinct phenotypes: first, the pollen produced was frequently morphologically abnormal and exhibited low viability; second, the anthers failed to dehisce and release pollen. Further mechanistic investigation revealed that *Ca*ZAT5 impedes anther dehiscence by suppressing the expression of genes associated with cell wall degradation. Consequently, the combined effects of reduced pollen quality and failure of pollen release resulted in a significant reduction in fruit yield. These findings provide a critical foundation for generating new male sterile lines in pepper and other crops.

## Introduction

Pepper (*Capsicum annuum* L.), a horticultural crop of the Solanaceae family, is the world’s widely cultivated spice crop and condiment [1]. F1 hybrid varieties generally exhibit enhanced yield and quality relative to their parents, making heterosis exploitation a central strategy in pepper breeding [2,3]. Current hybrid seed production methods for peppers mainly include manual emasculation, cross-hybridization, and male sterile line hybridization. Hybridization following manual emasculation ensures pollination but has high technical requirements, is labor-intensive, costly, and often results in low seed purity [4,5]. Therefore, male sterile lines are often used to reduce labor costs and improve seed purity, providing effective technical support for the efficient commercialization of hybrid pepper production [6].

Male sterility (MS) can be classified into genic male sterility (GMS) and cytoplasmic male sterility (CMS) [3,7]. GMS is mainly caused by structural or functional variations in nuclear genes and follows Mendelian inheritance patterns [8]. In contrast, CMS is a maternally inherited trait, caused by interactions between nuclear male sterility genes (Ms) and cytoplasmic sterility (S) genes, resulting in sterile offspring. Fertility restoration in CMS is achieved through nucleo-cytoplasmic interactions between restorer genes and S-cytoplasmic genes [9,10]. In pepper, several candidate CMS-associated genes, such as *orf300a* and *orf314a*, have been identified [11]. Additionally, various male sterile materials, including the CMS line FS1030A [12], sterile material 1A [13], and the GMS mutant msc-3 [14], have been characterized. Both CMS and GMS systems have been successfully applied in hybrid pepper production, yielding substantial agronomic, economic, and social benefits [4,14].

In addition, a distinct type of MS, known as functional MS, has been identified in mutants that produce normal pollen, but their anthers fail to dehisce or have abnormal morphology, resulting in incomplete dehiscence [15,16]. Unlike other MS types, functional MS does not require maintainer lines for hybrid seed production, and its pollen can be used for subsequent fertility restoration [17,18]. Anther dehiscence is a complex, tightly regulated process, where mutations or overexpression of key genes can lead to abnormal anther development, resulting in MS [19]. In *Arabidopsis*, the loss of function of *NST1* and *NST2* results in the complete absence of secondary thickening in the endothecium, leading to anther indehiscence [20], a phenotype similar to that observed in mutants of their upstream regulator, *MYB26* [18]. Likewise, the disruption of polygalacturonase genes *ADPG1* and *ADPG2* in Arabidopsis also causes indehiscence [21]. This regulatory mechanism is conserved in tomato, where loss of function of the PG gene (*PS-2*) directly leads to non-dehiscent anthers and functional male sterility [22]. Conversely, in rice, loss of *OsTIE1* function induces premature anther dehiscence [17], underscoring the necessity of precise temporal regulation of this process. Despite these advances in model plants, the genetic and molecular mechanisms underlying anther dehiscence in pepper (*Capsicum annuum*) remain largely unexplored.

Furthermore, Cys2/His2 zinc finger proteins (C_2_H_2_-ZFPs), one of the largest transcription factor families, are widely involved in the transcriptional regulation of flowering induction, floral organ development, and pollen and pistil maturation [23,24]. In rice, the C_2_H_2_-ZFP *SIP1* can specifically target *Early heading date 1* (*Ehd1*), altering H3K4me3 levels to promote flowering [24]. Silencing the meiosis-related ZFP 1 (*MEZ1*) in Arabidopsis causes abnormal male meiosis, resulting in meiocytes with variable chromosome numbers and DNA contents [25]. In Arabidopsis, C_2_H_2_-ZFPs such as *DUO1* and *DAZ1*/*DAZ2* also play key roles in pollen development, particularly in reproductive cell differentiation [26]. Similarly, in petunia, silencing the tapetum-specific ZFP *TAZ1* causes abnormal tapetum development and premature degeneration, microspore sterility, limited pollen grain production, and defective pollen walls [27]. In rapeseed, the C_2_H_2_-ZFP *BcMF20* regulates tapetal development and pollen viability [28]. Despite these findings, the functional roles of C_2_H_2_-ZFPs in regulating anther dehiscence and pollen release remain insufficiently characterized, and the genes associated with anther dehiscence in pepper are yet to be elucidated.

SUPPRESSOR OF OVEREXPRESSION OF CONSTANS 1 (SOC1) functions as a central regulator in the plant flowering pathway, promoting flowering [29]. As a typical MIKC-type MADS-box transcription factor, *SOC1* mediates the convergence of multiple environmental and endogenous cues, including photoperiod, ambient temperature, vernalization, and hormones, to precisely control the floral transition [30]. Its expression is regulated by a complex upstream cascade, including indirect positive regulation via CONSTANS (CO) through activation of FLOWERING LOCUS T (FT), and direct transcriptional repression by FLOWERING LOCUS C (FLC), which binds to its promoter [31–33]. Although the regulatory network involving SOC1 has been extensively characterized in model plants, the role of C_2_H_2_-ZFPs in modulating flowering time in pepper remains largely unexplored. In this study, we isolated the C_2_H_2_-ZFP transcription factor, *CaZAT5,* from pepper. Previous studies have demonstrated that low temperatures suppress its expression, implicating. *CaZAT5* acts as a negative regulator of cold tolerance in pepper. During phenotypic analysis of flowering and fruiting in T_0_ generation *CaZAT5*-overexpressing (OE) tomato, we unexpectedly observed phenotypes suggesting a potential role in floral induction and MS. Therefore, *CaZAT5* was selected for detailed functional investigation.

This study addresses the following research key questions: (1) What is the role of *CaZAT5* in regulating flowering time in pepper? (2) Which downstream genes are transcriptionally regulated by *CaZAT5* to influence flowering time? (3) How does OE *CaZAT5* overexpression affect anther development across different stages? (4) What are the structural and functional impacts of *CaZAT5* overexpression on pollen? (5) What mechanisms underlie the reduced fruit set rate and yield in *CaZAT5*-OE plants? The findings will provide new theoretical insights for the development of hybrid male sterile plants. This strategy, which focuses on regulating male fertility via pollen release rather than pollen production, may represent a new avenue for future hybrid breeding programs.

## Results

### Characterization of the *CaZAT5* sequence

**Fig 1:**
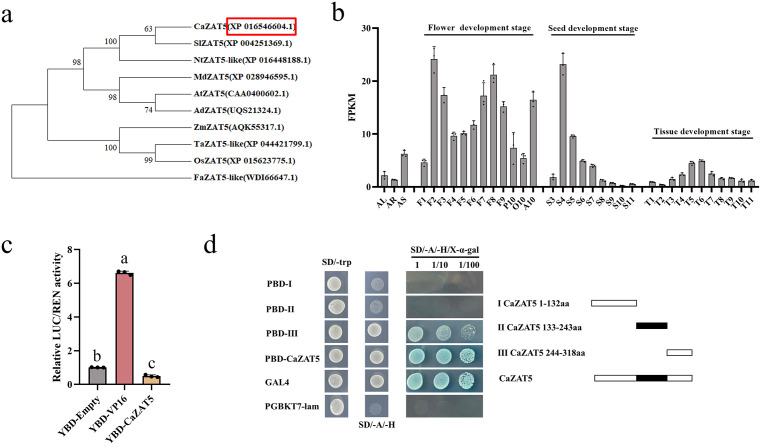
Characterization of the *CaZAT5* sequence. (a) Phylogenetic analysis of ZAT5. The phylogenetic tree was constructed using MEGA7 software with the neighbor-joining method and 500 bootstrap replications. Ca, *Capsicum annuum*; Sl, *Solanum lycopersicum*; Nt, *Nicotiana tabacum*; At, *Arabidopsis thaliana*; Zm, *Zea may*s; Os, *Oryza sativa*; Ta, *Triticum aestivum*; Fa, *Fragaria ananassa*. (b) Expression profile of *CaZAT5* in different tissues and developmental stages. The cartoon diagram of different developmental stages and tissue locations is shown in S1 Fig (c) Transcriptional activation assay of *CaZAT5*. Values represent the mean ± SD. (n = 3). ANOVA was performed using Duncan’s test, different letters mark significant differences (*P* < 0.05). (d) transcriptional activation assay in yeast. The right panel shows different fragments of *Ca*ZAT5 used to detect transcriptional activity.

Phylogenetic analysis of the ZAT5 protein family revealed that *Ca*ZAT5 (XP_016546604.1, pepper) clusters within the same subgroup as *Sl*ZAT5 (XP_004251369.1, tomato) and *Nt*ZAT5 (XP_016448188.1, tobacco), exhibiting the closest phylogenetic relationship to *Sl*ZAT5 (Fig 1a). In tomato, *SlZAT5* regulates multiple downstream target genes involved in reproductive growth and delayed fruit maturation, and softening [34]. These findings prompted further investigation into the role of *CaZAT5* in reproductive development. The full-length cDNA of *CaZAT5* contains a 954 bp open reading frame (ORF), encoding 318 amino acids. Each amino acid sequence includes two conserved ZnF_C_2_H_2_-type domains (located at positions 133–155 and 218–243), indicating that *CaZAT5* is a typical C_2_H_2_-ZFP transcription factor.

Expression data, involving roots, stems, leaves, flowers, seeds, and different developmental stages, derived from public pepper transcriptomic databases (source: http://pepperhub.hzau.edu.cn/index.php) revealed elevated *CaZAT5* expression in the flower development stage (S1 Fig) [35]. *CaZAT5* expression was higher during the early seed development phase, peaking at the S4 stage (Fig 1b). Therefore, *CaZAT5* may play a role in floral organ development in pepper.

A Dual-LUC Assay revealed that the LUC/REN ratio in tobacco leaves carrying pBD-CaZAT5 was significantly lower than the negative control (YBD) (Fig 1c). Yeast two-hybrid (Y2H) assays using the *CaZAT5* CDS confirmed that only yeast cells transformed with the full-length CaZAT5 ORF sequence (1–318 aa) and the CaZAT5-C-terminal sequence (244–318 aa) could grow normally on double-dropout medium (SD/-A/-H/X-α-gal) (Fig 1d). These results indicate that the CaZAT5 protein functions as a transcriptional repressor, with its C-terminal region (244–318 aa) responsible for transcriptional inhibition.

### *CaZAT5* negatively regulates flowering time

**Fig 2:**
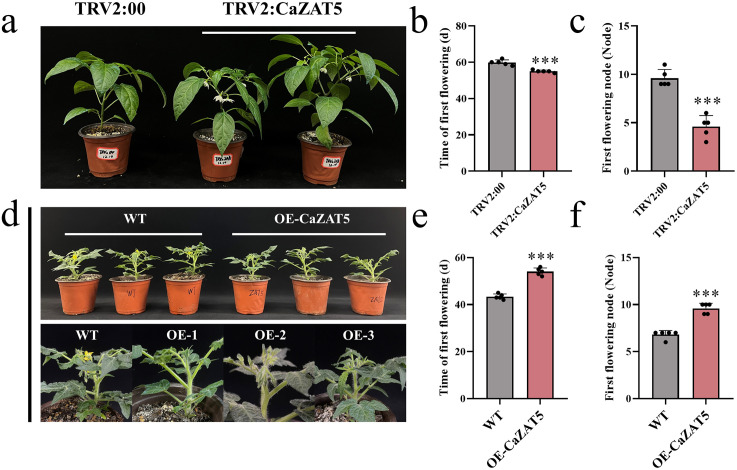
*CaZAT5* negatively regulates flowering time. (a) Phenotypic comparison of silenced *CaZAT5* promotes pepper flowering. (b) Flowering time of TRV2:00 and TRV2:CaZAT5 plants. (c) First flowering node of TRV2:00 and TRV2:CaZAT5 plants. (d) OE *CaZAT5* delays tomato flowering. (e) Flowering time of WT and OE *CaZAT5* tomato plants. (f) First flowering node of WT and OE *CaZAT5* tomato plants. Values represent the mean ± SD. (n = 5). *** indicating highly significant differences (*P* < 0.001).

To investigate the function of *CaZAT5* in pepper, we performed virus-induced gene silencing (VIGS). The positive control plants (TRV2:CaPDS) displayed the expected leaf bleaching phenotype at 21 days post-infiltration (S2a Fig). *CaZAT5* expression in TRV2:*Ca*ZAT5 plants was significantly lower than in the TRV2:00 control plants (S2b Fig). During flowering, TRV2:CaZAT5 plants exhibited apparent phenotypic differences compared to TRV2:00 plants (Fig 2a). Specifically, *CaZAT5*-silenced plants flowered significantly earlier and possessed a significantly lower first flowering node (defined as the true leaf node count between the cotyledons and the first main-stem flower) (Fig 2b, 2c). Under normal growth conditions, TRV2:CaZAT5 plants initiated flowering at 53–56 days, whereas TRV2:00 controls flowered at 58–62 days (Fig 2b).

In addition, we generated CaZAT5-overexpressing (OE) transgenic tomato lines under the control of the 35S promoter. PCR and RT-qPCR analyses confirmed the successful generation and significant overexpression of *CaZAT5* in six independent transgenic lines compared to wild-type (WT) plants (S2c, d Fig), which subsequently led to notable phenotypic differences. Phenotypic evaluation revealed that, while WT plants initiated flowering, *CaZAT5* OE1 and OE3 plants had not yet developed floral buds (Fig 2d). Moreover, *CaZAT5-*OE plants exhibited significantly delayed flowering, with an average flowering time of 52–56 days, approximately 10.4 days later than the WT plants (42–45 days), while the first flowering node was delayed by 2.8 nodes (Fig 2e, 2f). These observations confirm that *CaZAT5* functions as a negative regulator of flowering.

### *Ca*ZAT5 directly suppresses *CaSOC1* expression

**Fig 3:**
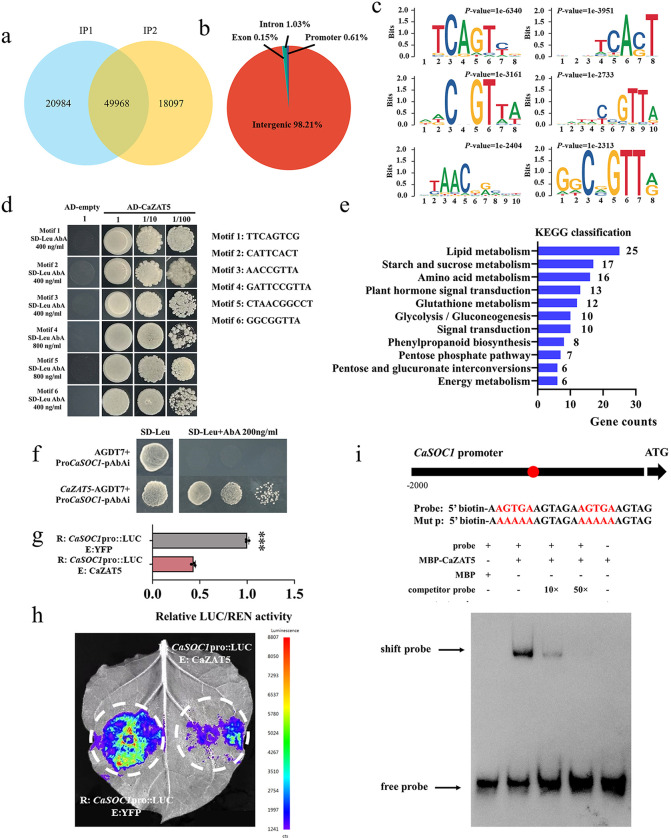
*Ca*ZAT5 directly suppresses *CaSOC1* expression. (a) Venn diagram of peak overlap within the group. (b) Distribution of reads across various genomic elements. (c) Binding motifs of the *Ca*ZAT5 protein identified by DAP-seq. (d) Y1H validation of the interaction between *Ca*ZAT5 and the binding motifs. (e) KEGG pathway enrichment analysis of downstream target genes identified by DAP-seq. (f) Y1H results. (g) LUC analysis results. Values represent the mean ± SD. (n = 3). *** indicating highly significant differences (*P* < 0.001). (h) *In vivo* imaging of the Dual-LUC assay. (i) Detected using EMSA was the specificity of the binding between *Ca*ZAT5 and the TCACT element in the *CaSOC1* promoter. “+” and “-” represent different combinations of lanes. The MBP protein was used as a negative control.

To investigate the molecular basis of *CaZAT5*-mediated delay in flowering, we used DNA affinity purification sequencing (DAP-seq) to analyze the potential binding sites of *Ca*ZAT5 across the pepper genome. Comparative analysis between biological replicates (IP *Ca*ZAT1 and IP *Ca*ZAT2) identified 49,968 peaks (Fig 3a). The binding sites were distributed as follows: 0.15% in gene exon regions, 1.03% in intron regions, 0.61% in promoter regions, and 98.21% in intergenic regions (Fig 3b). To investigate the potential direct targets of *Ca*ZAT5 that impact downstream gene transcription, we focused on genes with *Ca*ZAT5 binding sites located in their promoter region. A total of 370 genes were considered potential downstream target genes of *Ca*ZAT5 (S1 Table). Motif enrichment analysis using MEME identified six highly conserved cis-elements preferentially bound by *Ca*ZAT5 (Fig 3c). Y1H was subsequently performed to validate *Ca*ZAT5 binding to the six motifs *in vitro* (Fig 3d). KEGG enrichment analysis was subsequently performed to assess the functions of the putative target genes of *Ca*ZAT5. The identified target genes were predominantly enriched in metabolic pathways involving lipid metabolism, starch and sucrose metabolism, amino acid metabolism, plant hormone signal transduction, and glutathione metabolism (Fig 3e).

SOC1 is a well-established positive regulator and key integrator in the plant flowering-time network [29,30]. *CaSOC1* was identified by DAP-seq as a putative *Ca*ZAT5 target and was selected for further validation. Notably, a C_2_H_2_ binding site (1103–1113 bp: AAGTGAAGTAG) was identified in the *CaSOC1* promoter. A Y1H assay subsequently confirmed that *Ca*ZAT5 could bind to the C_2_H_2_ binding site in the *CaSOC1* promoter (Fig 3f). A Dual-LUC assay revealed that the LUC/REN ratio in tobacco leaves infiltrated with 35S:CaZAT5 and CaSOC1pro-LUC was significantly lower than that in leaves infiltrated with 35S: empty and CaSOC1pro-LUC. *In vivo* fluorescence imaging displayed the same result, confirming transcriptional repression (Fig 3g, 3h). EMSA assays demonstrated that the MBP-tagged *Ca*ZAT5 protein specifically interacted with the biotin-labeled *CaSOC1* promoter fragment (Fig 3g).

### Overexpression of *CaSOC1* accelerates floral transition and suppresses vegetative growth

**Fig 4:**
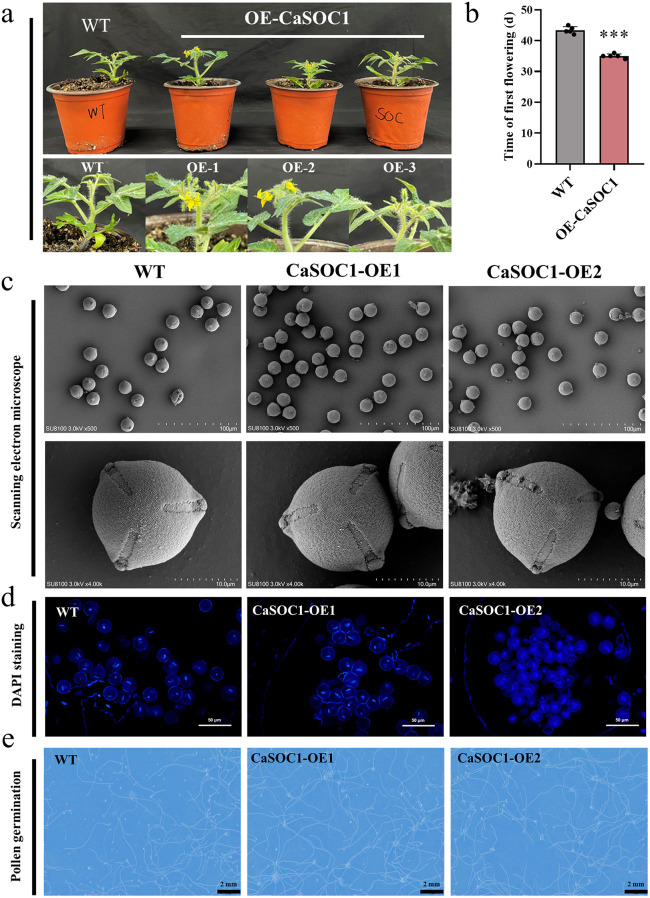
Overexpression of *CaSOC1* accelerates floral transition and suppresses vegetative growth. (a) Phenotype of overexpressing *CaSOC1* promotes tomato flowering. (b) Flowering time of WT and OE *CaSOC1* tomato plants. Values represent the mean ± SD. (n = 5). *** indicating highly significant differences (*P* < 0.001). (c) Scanning electron microscopy analysis of pollen grains in WT plants and OE *CaSOC1* plants (OE1 and OE2). Scale bars = 100 μm and 10 μm. (d) DAPI staining of pollen grains in WT plants and OE *CaSOC1* plants (OE1 and OE2). Scale bars = 50 μm. (e) *In vitro* germination test of pollen grains from WT plants and OE *CaSOC1* plants (OE1 and OE2). Scale bars = 2 mm. Values represent the mean ± SD. (n = 3). ANOVA was performed using Duncan’s test, different letters mark significant differences (*P* < 0.05).

We constructed *CaSOC1* OE tomato plants and identified five transgenic lines through RNA and RT-qPCR analysis for subsequent experiments to determine the biological function of *CaSOC1* (S3a, b Fig). *CaSOC1*-overexpressing plants flowered significantly earlier, with the average flowering time of the first flower occurring at 35 days (Fig 4a, 4b).

Scanning electron microscopy (SEM) revealed no discernible morphological differences in pollen grains between WT and *CaSOC1* OE1 and OE2 plants (Fig 4c). Subsequently, DAPI staining of mature pollen revealed that both OE1 and OE2 ultimately exhibited a normal uninucleate state (Fig 4d). *In vitro* pollen germination assays indicated enhanced viability in OE1 (88.67%) and OE2 (93.20%) compared to WT (80.31%) (Figs 4e and S3i). These results suggest that *CaSOC1* overexpression induces early flowering in tomato and enhances the pollen germination rate.

At the fruit maturity stage (140 days after sowing), *CaSOC1-*OE plants showed significantly reduced vegetative growth, plant height, and aboveground biomass (S3c–e Fig) compared to WT, while overall fruit yield remained unaffected (S3f Fig). Meanwhile, mature *CaSOC1-*OE fruits (length 22.05-24.5 mm, width 20.18-23.1 mm) were significantly larger than those of WT fruits (length 21.16 mm, width 18.22 mm) (S3g, h Fig). Thus, based on our findings, *CaSOC1* overexpression promotes reproductive growth (flowering and pollen viability) while inhibiting vegetative growth.

### *CaZAT5* overexpression promotes vegetative growth but reduces fruit set rate and yield

**Fig 5:**
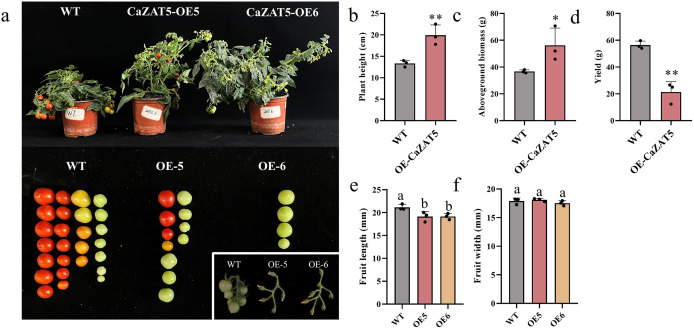
*CaZAT5* overexpression promotes vegetative growth but reduces fruit set rate and yield. (a) Phenotype of WT and OE *CaZAT5* plants (OE5 and OE6) during the fruiting period. The bottom right shows the flower sterility phenotype of OE *CaZAT5* plants. (b) Plant height. (c) Aboveground biomass. (d) Yield. (e) Fruit length. (f) Fruit width. Values represent the mean ± SD. (n = 3). * indicating highly significant differences (*P* < 0.05), ** indicating highly significant differences (*P* < 0.01). ANOVA was performed using Duncan’s test, different letters mark significant differences (*P* < 0.05).

*CaZAT5* overexpression significantly promoted vegetative growth in OE5 and OE6 plants, resulting in increased plant height and aboveground biomass (Fig 5a–5c). In WT plants, normal pollination resulted in 26 fruits with a fruit set rate of 76.47%. However, most OE5 and OE6 flowers displayed abnormal pollination, setting only 11 (16.92% fruit set rate) and 4 (5.71% fruit set rate) fruits, respectively, significantly reducing tomato fruit set and yield (Fig 5a, 5d). Meanwhile, *CaZAT5* overexpression significantly reduced fruit length but did not significantly affect fruit width (Fig 5e, 5f). These results suggest that *CaZAT5* overexpression promotes vegetative growth (plant height and aboveground biomass) but negatively impacts reproductive growth (fruit set rate and yield) in tomatoes.

### *CaZAT5* overexpression affects pollen development and reduces pollen viability

**Fig 6:**
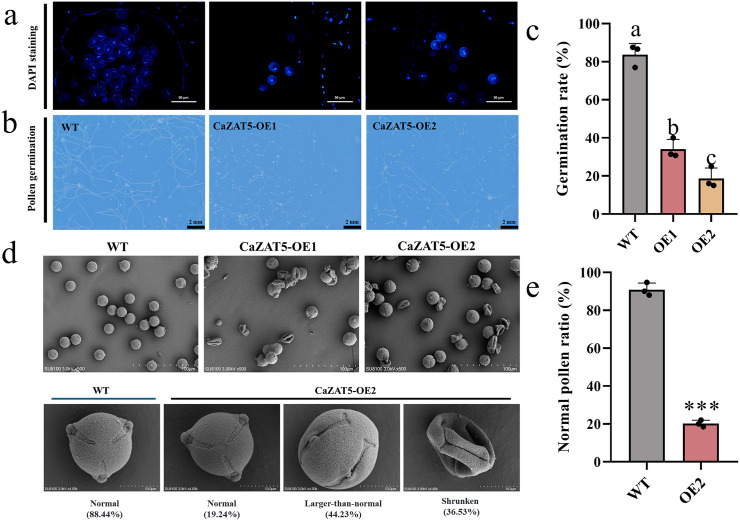
*CaZAT5* overexpression affects pollen development and reduces pollen viability. (a) DAPI staining of pollen grains in WT plants and OE *CaZAT5* plants (OE1 and OE2). Scale bars = 50 μm. (b) *In vitro* germination test of pollen grains from WT plants and OE *CaZAT5* plants (OE1 and OE2). Scale bars = 2 mm.(c) Pollen germination rate. Values represent the mean ± SD. (n = 3). ANOVA was performed using Duncan’s test, different letters mark significant differences (*P* < 0.05). (d) Scanning electron microscopy analysis of pollen grains in WT plants and OE *CaZAT5* plants (OE1 and OE2). Scale bars = 100 μm and 10 μm. (e) Normal pollen ratio. Values represent the mean ± SD. (n = 3). *** indicating highly significant differences (*P* < 0.001).

To further investigate the mechanisms underlying the low fruit set rate in *CaZAT5*-OE tomato plants, pollen morphology and viability were examined. DAPI staining of mature pollen revealed fewer mononucleate pollen grains in OE1 and OE2 compared to WT, with some pollen grains lacking a nuclear structure (Fig 6a). *In vitro* pollen germination assays revealed 34.08% and 18.66% germination rates of OE1 and OE2 pollen, respectively, significantly lower than WT pollen (Fig 6b, 6c). Furthermore, SEM analysis revealed that WT plants had 88.44% mature pollen grains (elliptical, with evenly distributed germination furrows). In contrast, mature *CaZAT5* OE2 pollen grains exhibited three phenotypes: normal, enlarged, and shrunken. Among them, irregular pollen grains accounted for 80.76%, comprising 44.23% enlarged (with abnormal germination furrows) and 36.53% shrunken and collapsed pollen grains. Normal pollen grains accounted for only 19.24% (Fig 6d, 6e). Therefore, the low fruit set rate in *CaZAT5-*OE plants is partly due to abnormalities in pollen grain development.

**Fig 7:**
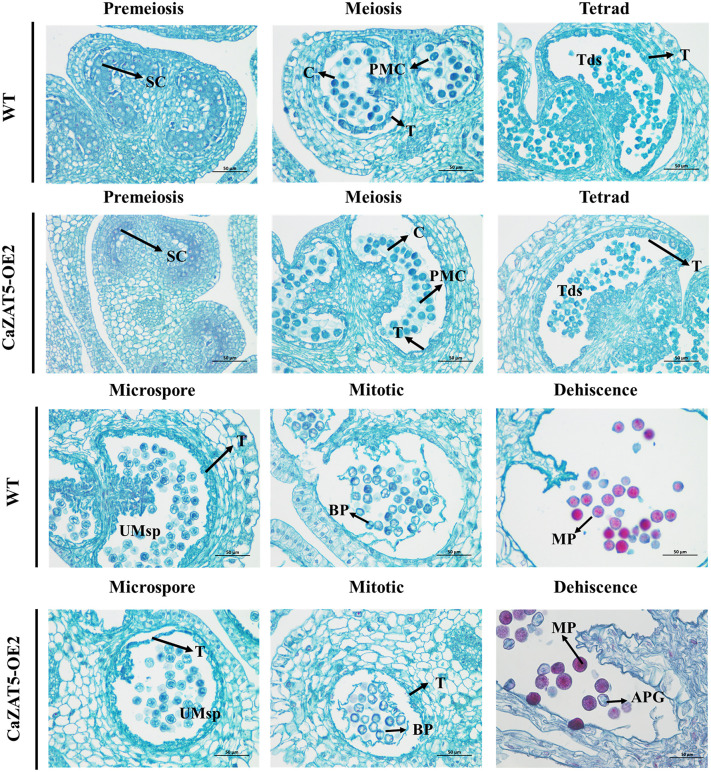
Safranin O-fast green staining was performed on sections of the six developmental stages of anthers in WT and *CaZAT5* OE2 tomato plants. The safranin O, with its strong affinity for DNA, bound to the DNA in the nucleus, resulting in red-stained granules. SC, sporogenous cell; C, callose; T, tapetum; Tds, tetrads; UMsp, uninucleate microspore; MP, mature pollen; BP, binucleate pollen; APG, abnormal pollen grain. Scale bars = 50 μm.

Histological analysis of anther development across six stages (from pre-meiosis to anther dehiscence) revealed no obvious abnormalities prior to meiosis in either WT or *CaZAT5* OE2 lines (Fig 7). Similarly, the SC of WT and *CaZAT5* OE2 anthers developed normally into PMC, which was surrounded by callose (Fig 7). During the tetrad stage, the callose in both WT and *CaZAT5* OE2 anthers degraded, gradually releasing the microspores. The tapetum cells in the WT anther began to break down and degenerate, disintegrating completely during the mitotic stage. At the mitotic stage, *CaZAT5* OE2 anthers exhibited a distinct phenotype, characterized by incomplete degradation of the tapetum, the presence of irregularly shaped and vacuolated microspores, and the apparent lack of nuclei in a subset of these cells. In the anther dehiscence stage, these abnormal microspores developed into irregularly shaped, swollen, and severely shriveled pollen grains. These results indicate that during pollen maturation, *CaZAT5* overexpression delays the degradation of tapetal cells, leading to abnormal pollen grain development.

### *CaZAT5* overexpression impairs normal pollen release in tomato

The fruit set rate is a direct indicator of plant fertility, which also depends on the development of the male and female reproductive organs [36]. *CaZAT5* overexpression affected pollen development, with only 19.24% of morphologically normal pollen grains observed in *CaZAT5* OE2 plants. Although this proportion was markedly reduced, *CaZAT5* overexpression might not be the sole reason for the reduced fruit. Indeed, histological staining on the longitudinal sections of anthers at six stages (pre-meiosis to anther dehiscence) showed no obvious phenotypic differences in the style, ovary, and ovules between *CaZAT5* OE2 and WT plants. *CaZAT5* OE2 and WT plants had normal ovaries and ovule numbers during the anther dehiscence stage (S4 Fig).

**Fig 8:**
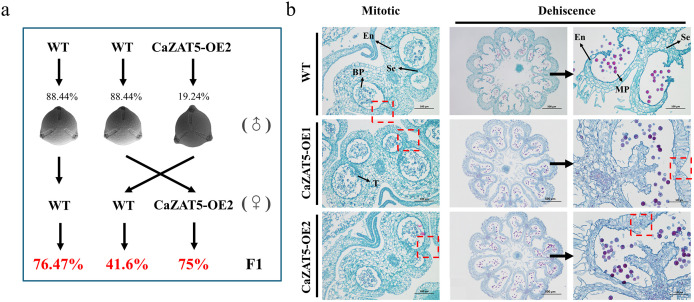
*CaZAT5* overexpression impairs normal pollen release in tomato. (a) Reciprocal cross experiments between *CaZAT5* OE2 and WT plants. ♂ represents male, and ♀ represents female. The WT plants and *CaZAT5* OE2 produce 88.44% and 19.24% normally shaped pollen grains, respectively. Red numbers represent the fruit set rate. (b) Safranin O-fast green staining of WT plants and OE *CaZAT5* tomato plants during the Mitotic and Dehiscence stages. The safranin O, with its strong affinity for DNA, bound to the DNA in the nucleus, resulting in red-stained granules. The red box indicates that the anther dehiscence in OE *CaZAT5* plants is impaired, preventing normal pollen release. En, endothecium; Se, septum; T, tapetum; MP, mature pollen; BP, binucleate pollen. Scale bars = 500 μm and 100 μm.

To test the fertility of pollen and female reproductive organs in *CaZAT5-*OE plants, we further conducted a reciprocal hybridization experiment between *CaZAT5-*OE and WT plants. As shown in Fig 8a, *CaZAT5* OE2 plants set fruit (75% fruit set rate) when pollinated with WT pollen. However, WT plants exhibited a substantially lower fruit set rate of 41.6% when pollinated with *CaZAT5* OE2 pollen. These results suggest that *CaZAT5* overexpression primarily impairs male fertility, while female reproductive function remains unaffected.

The fruit set rate from self-pollination in *CaZAT5-*OE plants was only 5.71–16.92% (Fig 5a), significantly lower than that observed in cross-pollinations between *CaZAT5* OE2 (♂) and WT (♀) hybridization (41.6%). These results suggest that pollen viability and other factors contribute to the decreased fruit set rate in self-pollinated *CaZAT5-*OE plants. Histological analyses of *CaZAT5* OE1 and OE2 plants at the pollen grain maturation stage (during mitosis and anther dehiscence) showed no significant differences in anther dehiscence between WT and *CaZAT5* OE1 and OE2 plants during mitosis (Fig 8b). In contrast, at the dehiscence stage, the septum and endothecium cells in the anthers of WT plants normally degrade and break down, forming a unilocular structure. The epidermal cells ruptured, and the anther opened normally, facilitating the release of pollen grains. In contrast, the septum and endothecium of *CaZAT5* OE1 and OE2 plants failed to degrade properly, preventing pollen grain release. Therefore, the failure of anther dehiscence in *CaZAT5-*OE plants is another major cause of sterility.

### *CaZAT5* overexpression alters the expression of cell wall degradation-related genes in tomato

Given that ZAT TFs are extensively involved in the transcriptional regulation of flower organ development, we hypothesized that *CaZAT5* influences pollen development and pollen release by modulating the transcription of downstream genes. We performed RNA-seq analysis on the leaf buds and anthers at the pollen maturation stage of the *CaZAT5-*OE tomato plants (S2 Table). Analysis of differentially expressed genes (DEGs) revealed that there was a total of 2285 DEGs between L-WT vs. L-CaZAT5, comprising 549 upregulated and 1736 downregulated genes, and 2799 DEGs between A-WT vs. A-CaZAT5, comprising 607 upregulated and 2192 downregulated genes. Venn analysis revealed 678 DEGs that were shared between the two comparison groups, L-WT vs. L-CaZAT5 and A-WT vs. A-CaZAT5 (S3 Table).

**Fig 9:**
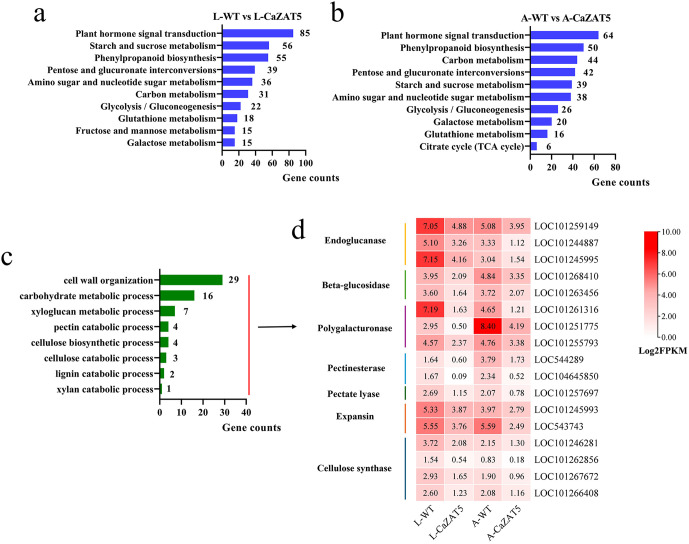
*CaZAT5* overexpression alters the expression of cell wall degradation-related genes in tomato. (a) KEGG classification annotation of DEGs in L-WT vs. L-CaZAT5. (b) KEGG classification annotation of DEGs in DEGs A-WT vs. A-CaZAT5. (c) Significantly enriched GO terms analysis of DEGs. (d) Expression profiling of DEGs related to cell wall relaxation and secondary wall thickening in WT and OE *CaZAT5* tomato.

Kyoto Encyclopedia of Genes and Genomes (KEGG) classification revealed that DEGs in both L-WT vs L-CaZAT5 and A-WT vs A-CaZAT5 were significantly enriched in multiple metabolic pathways, including plant hormone signal transduction, starch and sucrose metabolism, phenylpropanoid biosynthesis, pentose and glucuronate interconversions, carbon metabolism, and galactose metabolism (Fig 9a, 9b; S4 and S5 Tables). Further GO enrichment analysis showed that the shared DEGs (678 DEGs shared between L-WT vs L-CaZAT5 and A-WT vs A-CaZAT5) were also significantly enriched in cell wall metabolism-related processes, such as cell wall organization, carbohydrate metabolic process, xyloglucan metabolic process, pectin catabolic process, cellulose catabolic process and lignin catabolic process (Fig 9c). These findings suggest that *CaZAT5* overexpression may affect tomato anther dehiscence by regulating carbohydrate metabolism and lignin metabolism processes.

Anther dehiscence is a cell wall remodeling–dependent process involving the coordinated action of cell wall–degrading enzymes that catalyze the breakdown of pectin, cellulose, and lignin in the cell wall. Currently, several hydrolases and proteins associated with cell wall loosening have been confirmed to participate in this process [15,21,22]. In this study, *CaZAT5* overexpression significantly reduced the transcript levels of most genes in the cell wall metabolism pathway (Fig 9d). For instance, within the cellulose catabolism pathway, the transcript levels of endoglucanase (EG), which hydrolyzes glycosidic bonds, and beta-glucosidase (BG) were significantly suppressed in both leaves and anthers of *CaZAT5-*overexpressing tomato plants compared to the WT. The maximum downregulation observed for *EG* and *BG* was 2.93-fold and 2.26-fold, respectively (S3 Table). In carbohydrate and pectin catabolic pathways, CaZAT5 overexpression led to a pronounced decrease in the expression of pectin degradation-related genes, including polygalacturonase (PG), pectinesterase (PE), and pectate lyase (PL), in leaf buds and mature anthers. The transcript levels of *PG* were reduced up to 5.97-fold (S3 Table). We further observed that *CaZAT5* overexpression significantly reduced the expression level of expansin (EXP), a key gene involved in cell wall loosening and expansion, which plays a pivotal role in anther dehiscence [15,37].

Furthermore, anther dehiscence depends on the mechanical force provided by the secondary wall thickening of the endothecium [18,19]. Cellulose synthase (CESA) genes are indispensable for cellulose biosynthesis in the secondary wall [38]. Overexpression of *CaZAT5* significantly reduced the transcript levels of *CESA* in leaf buds and anthers, with the strongest repression observed in anthers (2.38-fold downregulation; S3 Table). RT-qPCR validation confirmed the RNA-Seq results (S5 Fig). These genes are associated with cell wall loosening, degradation, and secondary wall thickening, revealing that *CaZAT5* may influence tomato anther dehiscence by suppressing the transcript levels of genes involved in cellulose degradation, pectin degradation, cellulose synthesis, and expansin activity.

### *CaZAT5* suppresses cell wall loosening during anther dehiscence

**Fig 10:**
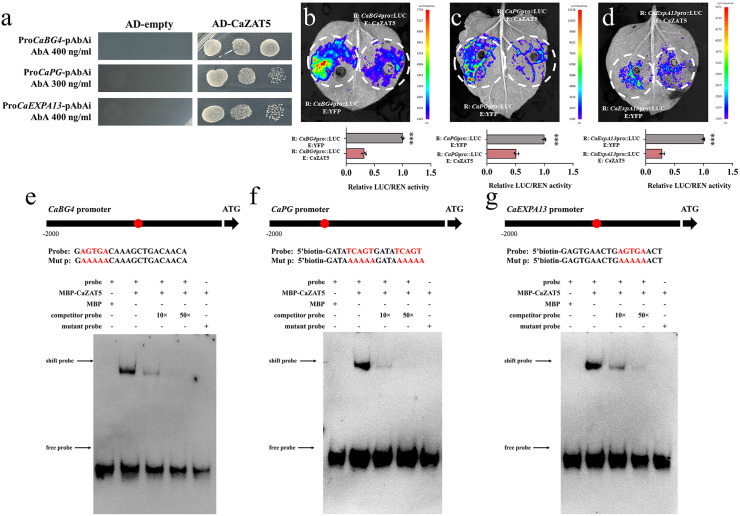
*CaZAT5* suppresses cell wall loosening during anther dehiscence. (a) Y1H results. (b-d) Dual-LUC results. Values represent the mean ± SD. (n = 3). *** indicating highly significant differences (*P* < 0.001). (e-g) Detected using EMSA was the specificity of the binding between *Ca*ZAT5 and the TCACT element in the *CaBG4*, *CaPG* and *CaEXPA13* promoter. “+” and “-” represent different combinations of lanes. The MBP protein was used as a negative control.

To identify direct downstream target genes of *Ca*ZAT5 associated with anther dehiscence, 370 candidate target genes were selected from DAP-seq analysis for further study (S1 Table), including xyloglucan endotransglucosylase2-like, polygalacturonases, β-1,3-Glucanases4, and expansin-A13. To validate the DAP-seq results, we performed Y1H assays, Dual-LUC assays, and EMSAs. Y1H assays demonstrated that *Ca*ZAT5 can bind to promoter elements of *CaBG4*, *CaPG*, and *CaExpA13 in vitro* (Fig 10a). Dual-LUC assays further revealed that co-expression of 35S:CaZAT5 with CaBG4pro-LUC, CaPGpro-LUC, and CaExpA13pro-LUC significantly reduced the LUC/REN ratio (by 67.52, 49.35, and 71.66%, respectively) compared to the control. Consistent with these results, in vivo fluorescence imaging showed markedly reduced luminescence in co-infiltrated tobacco leaves compared with the control group (Fig 10b–10d). Furthermore, EMSA confirmed that the *Ca*ZAT5 MBP recombinant protein binds to DNA probes containing the TCACT motif in the *CaBG4*, *CaPG,* and *CaExpA13* promoters (Fig 10e–10g).

In conclusion, *Ca*ZAT5 targets and regulates the promoter regions of *CaBG4*, *CaPG,* and *CaExpA13*, inhibiting their expression, thereby affecting cell wall loosening and degradation during anther dehiscence, ultimately influencing pollen release.

## Discussion

Cys2/His2-type ZFPs are widely involved in plant growth and development, including transcriptional regulation, abiotic stress, fruit ripening, and softening [39,40]. In Arabidopsis, *AtZAT5* negatively regulates pectin demethylesterification in seed mucilage, thereby ensuring proper mucilage formation [40]. *MdZAT5* maintains root development under drought stress and positively regulates drought tolerance in apples [41,42]. In tomato, *SlZAT5* regulates ethylene synthesis and inhibits fruit ripening and softening [34]. In kiwifruit, *AdZAT5* targets and regulates the expression of pectin-related genes, promoting fruit softening [43]. In this study, we characterized *CaZAT5* function during flowering and anther development by performing morphological, physiological, and molecular analyses on T_0_ generation *CaZAT5* OE tomato plants (used because of the difficulty in obtaining T_1_ progeny and the close phylogenetic relationship between SlZAT5 and CaZAT5). *CaZAT5* was shown to influence flowering time, pollen development, and anther dehiscence by regulating the expression of flowering and cell wall loosening genes, which are crucial for successful pollination in plants.

Flowering, a critical stage in the tomato lifecycle, marks the transition from vegetative to reproductive development. Accurate timing of flowering is essential for reproductive success and yield [44–46]. SOC1 is an important integrator of flowering signals, regulating floral meristem identity, morphology, and timing [29,47]. In many plant species, including *Brassica* [46], litchi [47], *Bambusa oldhamii* [48], and *Arabidopsis* [49], *SOC1* acts as a flowering activator promoting flowering. In this study, we identified a C_2_H_2_-ZFP transcription factor, *Ca*ZAT5, which is highly expressed during pepper floral development (Fig 1b). Functional analysis revealed that *Ca*ZAT5 knockdown led to early flowering in pepper, while its overexpression delayed flowering in tomato, underscoring its important role in regulating flowering time (Fig 2b, 2e). Using DAP-seq, we further identified *CaSOC1,* a flowering-promoting gene*,* as a potential downstream target of *Ca*ZAT5. Overexpression of *CaSOC1* in tomato promoted reproductive growth, manifested as early flowering and larger fruits. At the same a suppression of vegetative growth was observed, resulting in dwarfism and reduced shoot biomass, while pollen germination rate was increased (Figs 4a; S3d–S3i). Collectively, these findings demonstrate that *Ca*ZAT5 delays flowering by repressing the expression of *CaSOC1*.

Male sterility (MS) arises when anthers or pollen develop abnormally or exhibit functional defects [9,10]. These plants produce abnormal, non-viable pollens incapable of fertilization [15]. MS can be structural, sporogenic, or functional [16]. WT plants had a 76.47% fruit setting rate in this study, while *CaZAT5* OE lines (OE5 and OE6) plants showed drastically reduced rates of 16.92% and 5.71%, respectively (Fig 5a). Furthermore, SEM analysis revealed two abnormal pollen phenotypes in CaZAT5 OE plants: enlarged (44.23%) and shrunken pollen grains (36.53%) (Fig 6d, 6e). DAPI staining and pollen germination tests showed fewer single-celled nuclei and germinating pollen grains in OE *CaZAT5* (Fig 6a, 6b). Therefore, *CaZAT5* affects pollen development, leading to pollen sterility.

Cytological observations have demonstrated that pollen abortion in pepper primarily occurs during the tetrad stage [15,16]. Aberrant development and defective degradation of the tapetal cells disrupt the nutrient supply from callose to the developing microspores, preventing the proper separation of mature microspores from tetrads and ultimately resulting in pollen abortion [14,36]. In the CMS line FS1030A, male sterility occurs before tetrad formation, resulting in abnormal changes (such as excessive vacuolization and premature disintegration) in the tapetum cells, insufficient nutrient supply to microsporocytes, and ultimately sterility [12]. In the 1A line, MS occurs after the onset of the tetrad stage, where abnormal expansion causes compression and rupture in tetrad cells, interfering with normal microspore development and causing sterility [13]. In the GMS mutant, *msc-3*, abnormal degradation and vacuolization of tapetum cells delay tetrad formation, blocking microspore release and causing sterility [14]. In this study, the tapetum of *CaZAT5-*overexpressing anthers failed to undergo complete degradation during microsporogenesis (Fig 7). Consequently, the haploid microspores developed vacuolated and enucleated structures. At the dehiscence stage, a combination of swollen and shrunken pollen grains was observed. These results indicate that *CaZAT5* regulates tapetal degradation during microsporogenesis, and its overexpression disrupts this process, ultimately impairing pollen grain development.

Additionally, several genes regulating carbohydrate metabolism also affect plant fertility [36]. Carbohydrates are key components of the anther and are crucial in maintaining cell structure, providing energy, and supporting male fertility [36,50]. For instance, glucose, the predominant sugar in the anther, acts as a key energy source during pollen germination [51]. Deficiency in glucose or starch content within the anther severely impairs pollen development, leading to MS. In rice, the *csa* mutant exhibits reduced carbohydrate content in the later stages of anther development, resulting in MS [52]. In tomato, loss of *SlMYB33* function restricts the expression of starch metabolism-related genes in the anther, removing the necessary nutritional reserves during pollen maturation and causing pollen sterility [53]. *SlPIF4* overexpression delays tapetum degradation, significantly reducing the levels of Glc and Fru in the anther, ultimately causing abnormal pollen development [54]. In tobacco, downregulation of *CWIN* disrupts starch biosynthesis and compromises cell wall integrity, leading to pollen sterility [51]. Transcriptomic analysis revealed that the DEGs in the OE *CaZAT5* anthers were significantly enriched in metabolic processes related to starch and sucrose metabolism, pentose and glucuronate interconversions, carbon metabolism, fructose and mannose metabolism, and galactose metabolism, compared to WT plants (Fig 9a, 9b). Therefore, *Ca*ZAT5 may impair pollen development and fertility through the suppression of carbohydrate metabolic processes.

Normal pollen release from the anther is a key step in the sexual reproduction of flowering plants, and anther dehiscence is a complex, multi-stage process [19]. Before dehiscence, the septum between the anther chambers degrades, forming a bilocular anther [15,16]. The cells in the longitudinal region of the septum (the ‘stomium’) rupture, while lignin is deposited in the cell walls around the epidermal cell layer, generating an inward expansion force that ruptures the stomium, releasing pollen. Although this mechanism typically ensures efficient pollen dispersal, failure of anther dehiscence leads to functional MS [15,17]. In tomato, spontaneous mutations such as *ps* and *ps2* result in indehiscent anthers and functional MS [22]. Genes such as *SAF1* [55], *NST1*/*2* [56], and *AHP4* [57] also affect anther dehiscence in Arabidopsis. In this study, OE *CaZAT5* tomatoes set normal fruits after pollination with WT pollen (CaZAT5♀ × WT♂), with a 75% fruit set rate. Meanwhile, WT plants pollinated with OE *Ca*ZAT5 pollen (WT♀ × CaZAT5♂) displayed a 41.6% fruit set rate (Fig 8a), higher than the self-pollination fruit set rate (*Ca*ZAT5♀ × *Ca*ZAT5♂) (OE5 and OE6 fruit set rates were 16.92 and 5.71%, respectively). Longitudinal anther cross-sections at different developmental stages revealed normal pistil development (S4 Fig). Further tissue analysis revealed that at dehiscence, the septum and endothecium of *CaZAT5-*overexpressing tomato anthers degraded improperly, causing the anther pores to remain closed and preventing normal pollen release (Fig 8b). These results suggest that decreased pollen viability and impaired pollen release caused a low fruit set rate and reduced yield in *CaZAT5-*overexpressing plants.

An in-depth analysis of the carbohydrate-related DEGs further revealed that *CaZAT5* OE markedly suppressed the expression of cell wall degradation genes in anthers, including endoglucanase (*EG*), beta-glucosidase (*BG*), polygalacturonase (*PG*), pectinesterase (*PE*), and pectate lyase (*PL*) (Fig 9d). These hydrolytic enzymes are critical for the degradation of pectin and other polysaccharides in the anther cell wall, facilitating the breakdown of the middle lamella in the anther septum area [15,21,36]. Further experiments revealed that *Ca*ZAT5 directly regulates *CaPG* and *CaBG4* (Fig 10), contributing to cell wall loosening and separation processes, and promoting anther dehiscence. In Arabidopsis, three PGs (*ADPG1*, *ADPG2*, and *QRT2*) are involved in anther dehiscence [21], while a *PG* gene associated with anther dehiscence, *PS-2*, has been described in tomato. Mutations in the *PS-2* gene result in failed anther dehiscence in tomato [22]. Suppression of the β-glucanase gene *Osg1* in rice inhibits callose degradation during the tetrad stage, resulting in delayed microspore release and ultimately leading to pollen abortion [58]. Further, the rupture of the anther opening requires outward pressure from the anther wall, caused by the mechanical expansion force from the secondary thickening of the lignocellulose of the endothecium, which is crucial for providing the mechanical force necessary for anther dehiscence [37,59]. Disrupting the endothecium abolishes anther dehiscence, causing MS [59]. In Arabidopsis, *CsACS2* overexpression inhibits secondary cell wall thickening in transgenic plants, suppressing anther dehiscence and resulting in MS [60]. *NST1* and *NST2* in Arabidopsis also affect anther dehiscence by inhibiting secondary wall thickening [61]. In this study, *CaZAT5-*overexpresing plants exhibited downregulation of the key cellulose synthase gene *CESA* required for anther secondary wall synthesis (Fig 9d), and directly repressed the expansin gene *CaEXPA13* (Fig 10d). Expansins play a crucial role in regulating the mechanical properties of plant cell walls, promoting wall relaxation, extension, and rupture during anther dehiscence and pollen release [37,59]. These results suggest that *CaZAT5* may inhibit the normal septum and endothecium degradation by repressing *CaPG* and *CaBG4* expression. In contrast, the disruption of secondary wall cellulose synthesis and suppression of *CaEXPA13* expression lead to a lack of outward expansive pressure in the anther wall, preventing proper anther dehiscence.

**Fig 11:**
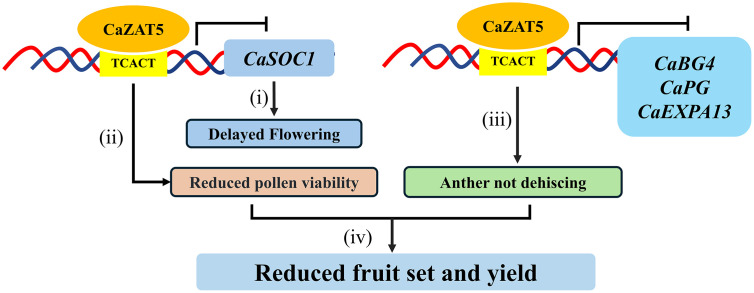
Working model of *CaZAT5* in flowering time and pollen development and release.

Based on these findings, we propose a functional model describing the regulatory role of *CaZAT5* in flowering time, pollen development, and release (Fig 11). In this model, (i) *CaZAT5* affects flowering time by inhibiting *CaSOC1* expression; (ii) *CaZAT5* suppresses carbohydrate metabolism, thereby impairing pollen development. (iii) *CaZAT5* affects anther dehiscence and pollen release by directly repressing *CaBG4*, *CaPG* and *CaEXPA13* expression. (iv) *CaZAT5* consequently reduces fruit set and yield by affecting pollen viability and release. These findings provide experimental evidence for understanding MS in peppers and establish a theoretical basis for the development of functional male-sterile lines for hybrid breeding.

## Materials and methods

### Phylogenetic tree

The target protein sequence (ZAT5) was obtained through BLAST (Basic Local Alignment Search Tool) on the NCBI website (https://www.ncbi.nlm.nih.gov/), and a phylogenetic analysis of ZAT5 was constructed using the Neighbor Joining (NJ) method in MEGA software (Pennsylvania State University, Tennessee, UAS).

### Transcriptional activation activity

Construct the reporter vector according to previous descriptions [62]. The obtained recombinant plasmids (pBD-CaZAT5, positive control pBD-VP16, and negative control pBD-Empty) were co-infiltrated into tobacco leaves with *A. tumefaciens* GV3101 containing the 5 × UAS-TATA-LUC plasmid. After dark incubation for 2 days, the LUC/REN ratio was measured.

The full-length CDS sequence of *CaZAT5* and three sequence fragments (N-terminal: 1–132 aa, ZnF-type conserved domain: 133–243 aa, C-terminal: 244–318 aa) were transformed into yeast strain Y2H and cultured on double-deficient (SD/ − Ade/ − His) media with or without X-α-gal. The colony growth was observed after three days of inverted incubation.

### Plant materials and growth conditions

Seeds of pepper, tobacco, tomato, and *Arabidopsis thaliana* were sown in nutrient soil and cultivated in a growth chamber with a daytime temperature of 25^o^C, a nighttime temperature of 20^o^C, and 16 hours of light.

### VIGs of *CaZAT5*

The VIGS experiment was conducted as described previously [62]. The *A. tumefaciens* GV3101, transformed with TRV2: CaZAT5 and TRV2: CaPDS, was mixed with *A. tumefaciens* transformed with TRV1 at a 1:1 (v/v) ratio and injected into the cotyledons of pepper seedlings. After 48 hours of dark treatment, the seedlings were moved to a growth chamber for further cultivation.

### The generation of transgenic tomato

The *CaZAT5* CDS was cloned into the PCAMBIA-EYFP vector and transformed into the *A. tumefaciens* GV3101 strain. For transgenic tomatoes, the leaf disc method was used for infection, and elongation and rooting were conducted on MS medium containing 50 mg/L hygromycin, ultimately generating T_0_ plants.

### Measurement of physiological indicators

The flowering time was evaluated using two criteria: first, the flowering node (The number of true leaf nodes between the cotyledons and the first flower on the main stem) and second, the time from sowing to the appearance of the first flower bud. Five plants were measured and counted for each time point. After 135 days of tomato sowing, plant height, fresh weight of the aboveground part, and yield parameters were recorded using a balance and ruler, and the fruit length and width were measured using a caliper. Each treatment had three biological replicates. WT plants and *CaZAT5* OE2 were used as male and female parents for the hybridization experiment, and the fruit set rate was statistically analyzed.

### Pollen germination experiment, phenotypic observation, and histological examination

Pollen germination experiments were conducted following Pei et al [36]. Pollen grains were collected and deposited on germination medium, and after incubating for 3 hours at 25°C, images were taken under a Leica microscope (Leica, Wetzlar, Germany) and the germination rate was calculated.

For DAPI staining, the method described by Wu et al. was followed [3]. Anthers from WT plants and *CaZAT5* OE2 plants were stained with 0.1 mg/mL DAPI, and nuclear status was assessed using an emission signal of 350nm/460nm (DM6 B, Leica, Wetzlar, Germany).

During the flowering period, flower buds at different developmental stages were collected for Safranin O-fast green staining. After embedding in paraffin and sectioning, the characteristics of the anther cells were examined using an Olympus microscope (Olympus BX51TRF, Olympus Corporation, Tokyo, Japan). The stages were classified as follows: Stage 1 (Premeiosis), bud diameter 1–2mm; Stage 2 (Meiosis), bud diameter 2.5-3.5mm; Stage 3 (Tetrad), bud diameter 3–4mm; Stage 4 (Microspore), bud diameter 5mm; Stage 5 (Mitotic), bud diameter 5–7mm; Stage 6 (Dehiscence), bud diameter > 7.5mm. Specific phenotypes are shown in S6 Fig.

For scanning electron microscopy, the method from Wu et al. was followed [3]. Flowers were fixed in a 2.5% glutaraldehyde solution (in phosphate buffer, pH 7.0) 24 hours at 4°C, and mature pollen was then fixed onto SEM stubs and coated with gold-palladium. Pollen morphology was observed using a scanning electron microscope (Hitachi S-4800, Tokyo, Japan).

### RNA-seq and RT-qPCR

Total RNA was extracted from four types of tomato tissues: leaf buds (L) and anthers at the pollen maturation stage (A, corresponding to the “Dehis” stage defined in Supplementary S6 Fig) of both wild-type (WT) and *CaZAT5* overexpressing transgenic plants (CaZAT5), using an RNA extraction kit (Thermo Electron, Waltham, USA). These samples are abbreviated as L-WT, A-WT, L-CaZAT5, and A-CaZAT5, respectively. Perform high-throughput sequencing on the Illumina Hiseq 2500 platform at Novogene Technology Co., Ltd. (Beijing, China). The criteria for differential gene expression were a Fold Change ≥ 2 and FDR < 0.01.

RT-qPCR was performed in 20 μL reaction volumes with 34 cycles, using *SlUBI3* as the internal reference. Relative expression was calculated by the delta-delta Ct (2^-ΔΔCT^) method. The primers used in this study are listed in S6 Table.

### DAP-seq and data analysis

The DAP-seq experiment was performed by Biorun (Wuhan, China). The experiment began with the extraction of genomic DNA (gDNA) from pepper leaves, which was then fragmented. Next, a DAP library was constructed, and a Halo-tagged CaZAT5 *in vitro* expression plasmid was created. Protein expression was carried out using the wheat embryo system. After the expressed CaZAT5 protein was bound to the gDNA library, affinity purification was used to isolate the specifically bound DNA fragments. Subsequently, multiple washes were performed to remove non-specifically bound chromatin, and high-purity DNA fragments were obtained for high-throughput sequencing analysis.

### Y1H, dual-LUC, and EMSA assays

The CDS of *CaZAT5* was cloned into the pGADT7 vector to construct the prey vector. The TCACT elements from the promoters of *CaSOC1*, *CaPG*, *CaXHT2-like*, *CaBG4* and *CaEXPA13* were cloned into the pAbAi vector to obtain the bait. The interaction between CaZAT5 and the promoter fragments was detected on SD/-Leu medium containing Aureobasidin A.

The promoters of *CaSOC1*, *CaPG*, *CaBG4* and *CaEXPA13* (2000 bp) were individually cloned into the pGreenII-LUC vector, and the CDS of *CaZAT5* was cloned into the pGreenII 62-SK vector as an effector. Subsequently, the transformed GV3101 was injected into tobacco leaves, and *in vivo* imaging and LUC/REN activity measurements were performed.

The recombinant CaZAT5 protein fused with the MBP tag was obtained using a prokaryotic expression system. DNA sequences of the promoter fragments labeled with 5’ biotin were used as probes. Next, gel EMSA was conducted following the guidelines provided in the EMSA chemiluminescent kit (Thermo Fisher Scientific, 20148, Waltham, USA).

### Accession numbers

The sequences of the following genes are available in the NCBI database: *CaZAT5*, XM_016691118.2; *CaSOC1*, XM_047395763.1; *CaPG*, XP_016571699.1; *CaBG4*, XM_016720615.2; *CaEXPA13*, XP_016570424.1.

### Statistical analysis

The data were analyzed using analysis of variance (ANOVA) in SPSS 22.0 (IBM, New York, USA). Figures were generated with GraphPad Prism 8 (GraphPad Software, California, USA). Statistical analysis results are presented in S7 Table.

## Supporting information

S1 Fig Cartoon expression profile of *CaZAT5* in different tissues and developmental stages. Different colors represent different gene expression levels (Log2 FPKM).
(TIF)

S2 Fig Identification of *CaZAT5* silencing and overexpression plants. (a) Phenotype of leaf bleaching in the positive control. (b) RT-qPCR identification of TRV2-CaZAT5.
(TIF)

S3 Fig Overexpression of *CaSOC1* suppresses vegetative growth in tomato. (a) PCR identification of transgenic tomato. (b) RT-qPCR identification of transgenic tomato. (c) Phenotypic comparison between WT and OE *CaSOC1* tomato plants at the fruit ripening stage. (d-h) Plant height, Aboveground biomass, Yield, Fruit length, and Fruit width of WT plants and OE *CaSOC1* plants.
(TIF)

S4 Fig Safranin O-fast green staining of longitudinal sections of flower buds at different developmental stages in WT and *CaZAT5* OE2 tomato plants. (TIF)

S5 Fig RT-qPCR analysis of the relative expression levels of DEGs. (TIF)

S6 Fig Phenotypes of tomato flower buds at different stages of flowering. (TIF)

S1 Table The predicted downstream target genes of *Ca*ZAT5. (XLSX)

S2 Table Transcriptome sequencing data analysis. (XLSX)

S3 Table There are 678 common DEGs between the two comparison groups, L-WT vs L-CaZAT5 and A-WT vs A-CaZAT5. (XLSX)

S4 Table KEGG classification annotation of DEGs in L-WT vs. L-CaZAT5. (XLSX)

S5 Table KEGG classification annotation of DEGs in DEGs A-WT vs. A-CaZAT5. (XLSX)

S6 Table Primers were used for the RT-qPCR. (XLSX)

S7 Table Statistical analysis results. (XLSX)
