# Supplementary figures and images for: CaZAT5 delays the flowering time in tomato and affects pollen viability and anther dehiscence
Source: PLoS Genet. 2026 Jan 6;22(1):e1012016. doi: 10.1371/journal.pgen.1012016 (PMC12788657; doi:10.1371/journal.pgen.1012016)

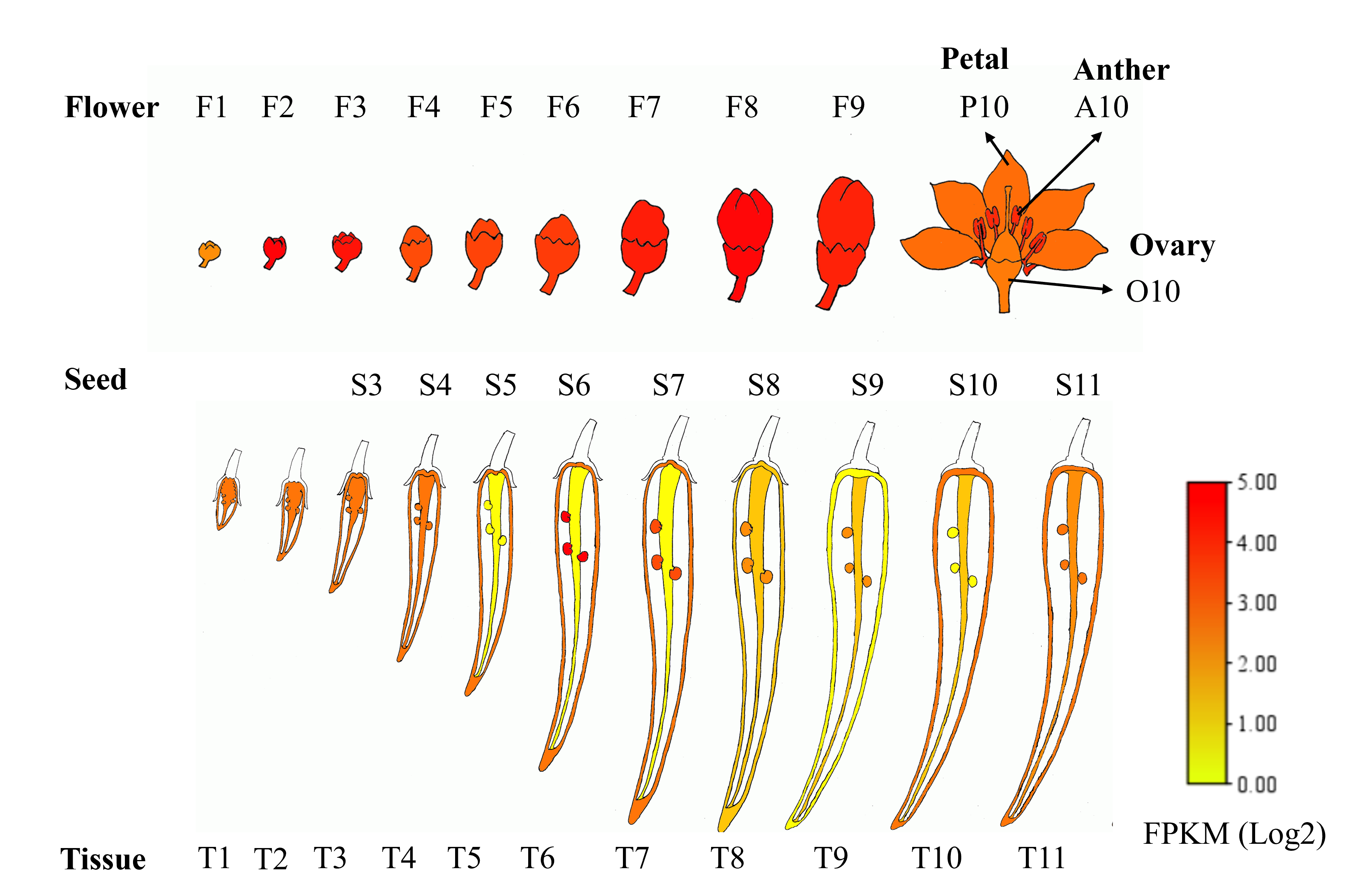

Supplement: S1 Fig — Different colors represent different gene expression levels (Log2 FPKM). (TIF) [file pgen.1012016.s001.tif]

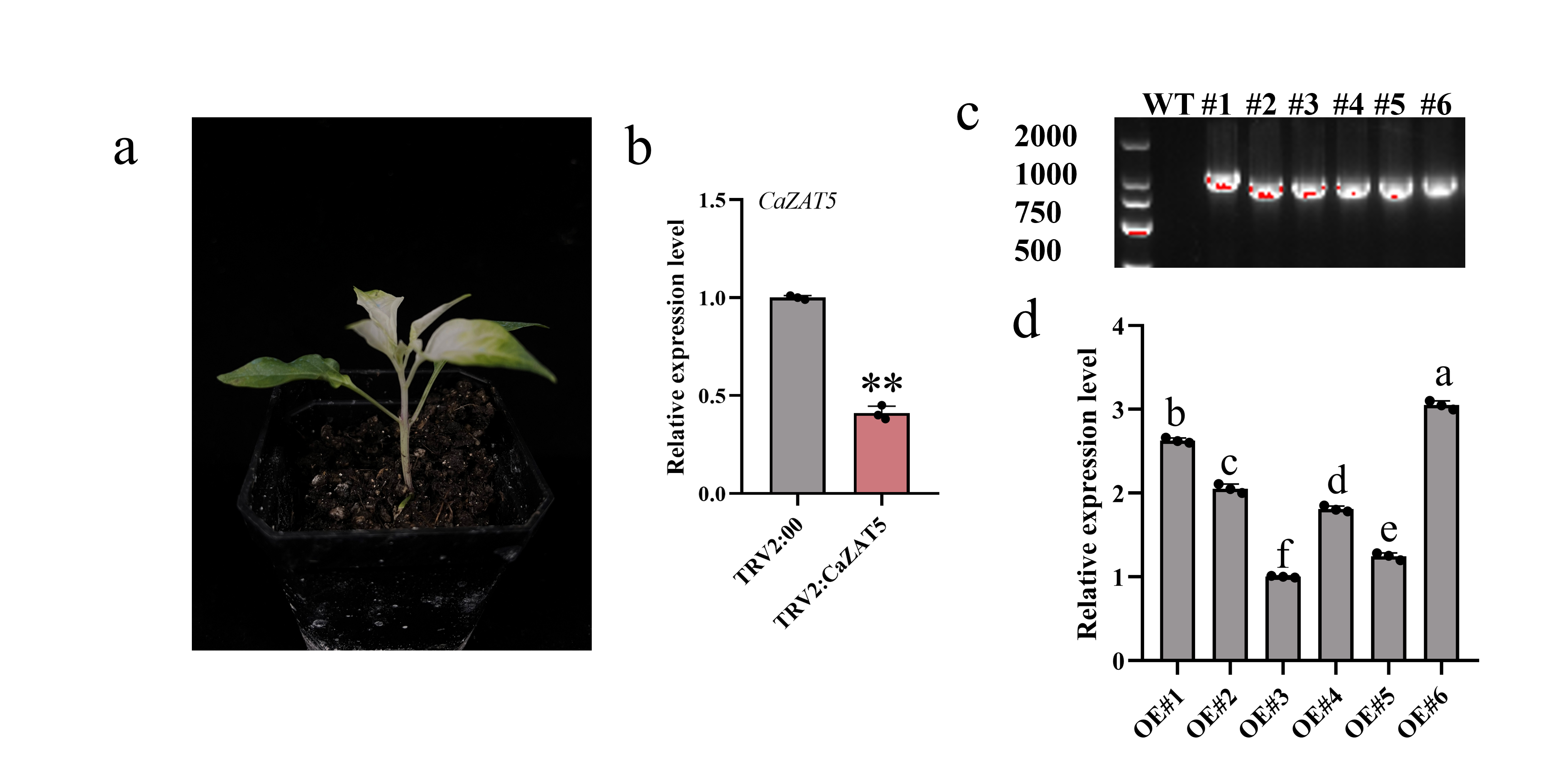

Supplement: S2 Fig — (a) Phenotype of leaf bleaching in the positive control. (b) RT-qPCR identification of TRV2-CaZAT5. (TIF) [file pgen.1012016.s002.tif]

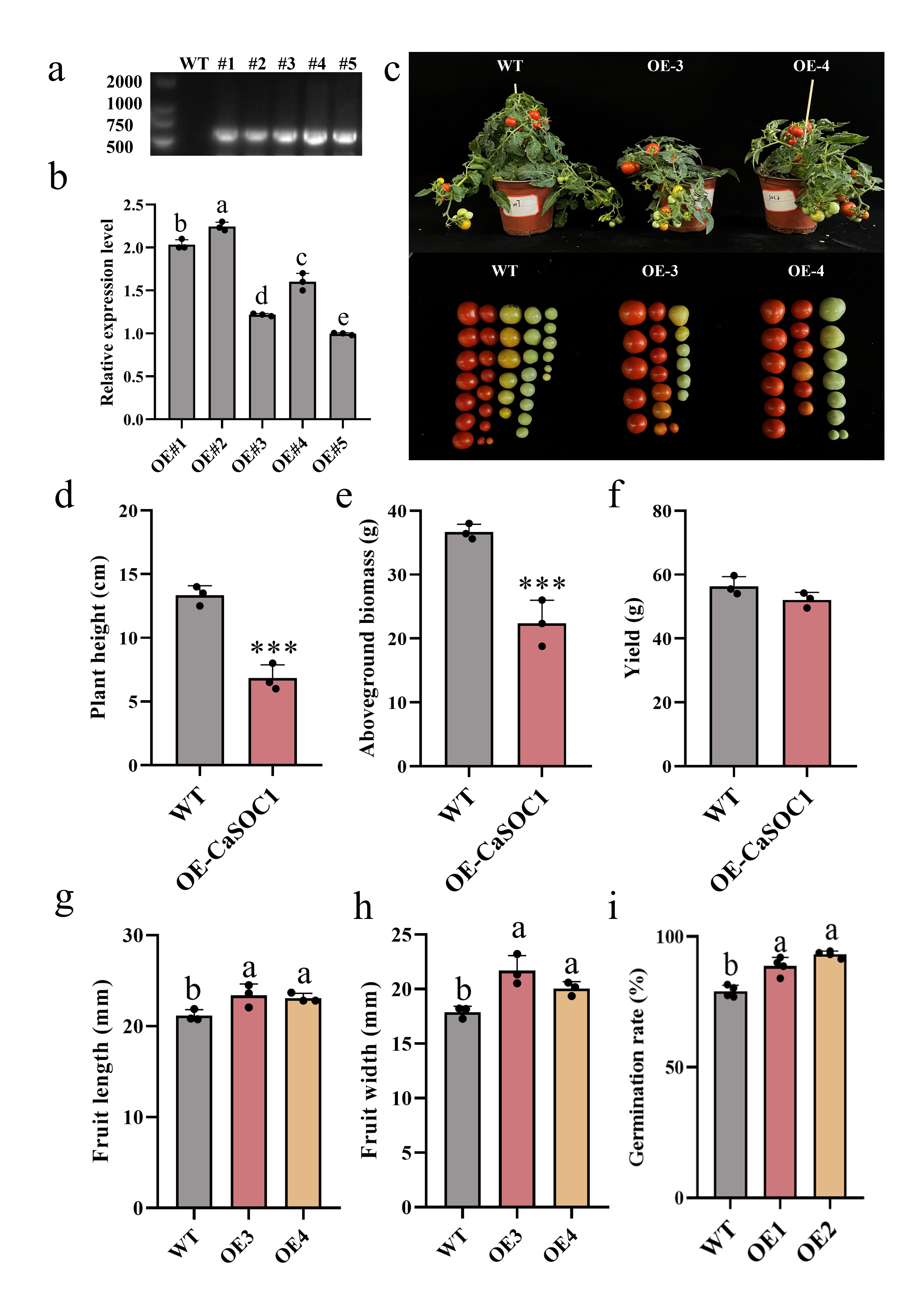

Supplement: S3 Fig — (a) PCR identification of transgenic tomato. (b) RT-qPCR identification of transgenic tomato. (c) Phenotypic comparison between WT and OE CaSOC1 tomato plants at the fruit ripening stage. (d-h) Plant height, Aboveground biomass, Yield, Fruit length, and Fruit width of WT plants and OE CaSOC1 plants. (TIF) [file pgen.1012016.s003.tif]

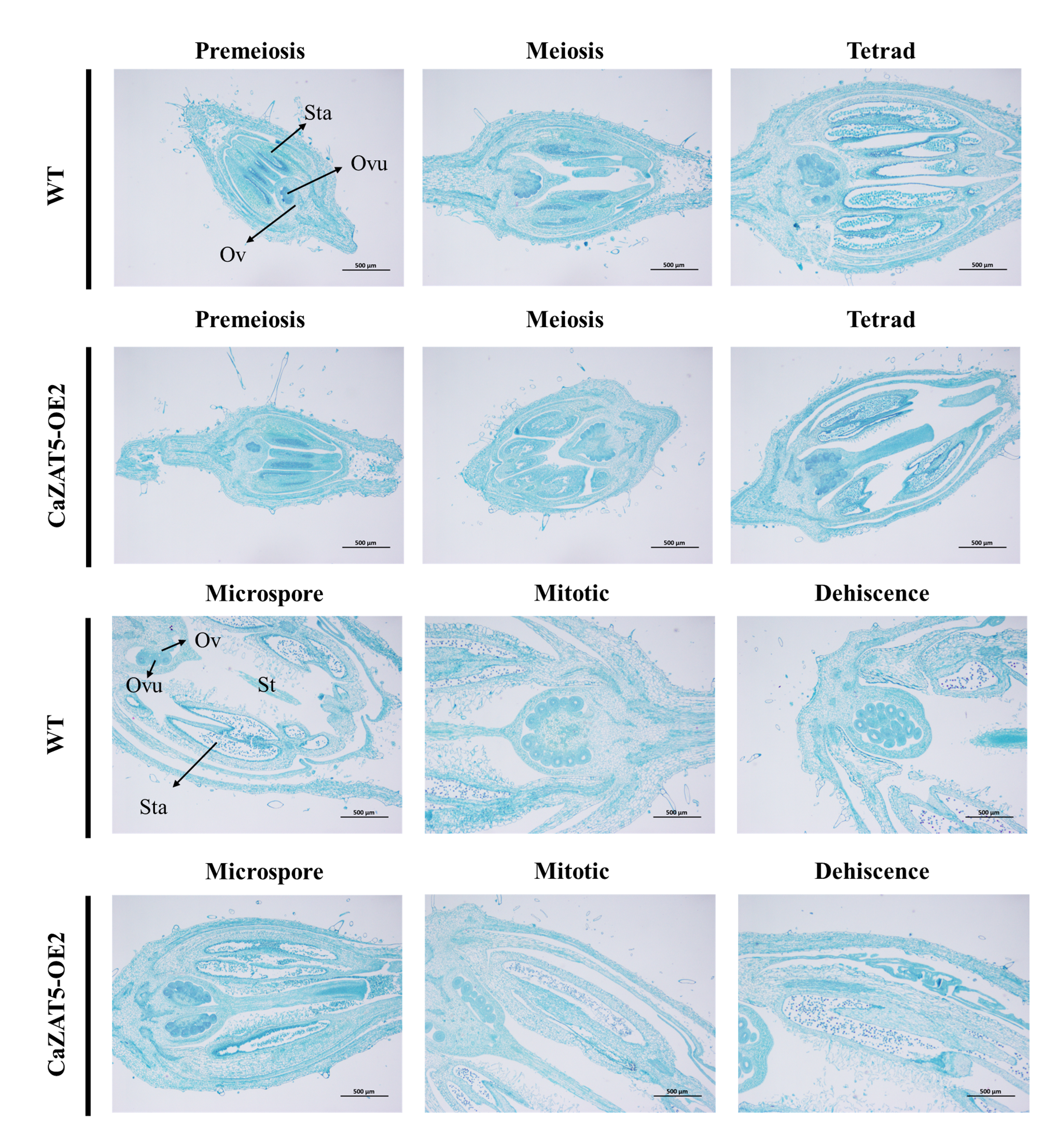

Supplement: S4 Fig — (TIF) [file pgen.1012016.s004.tif]

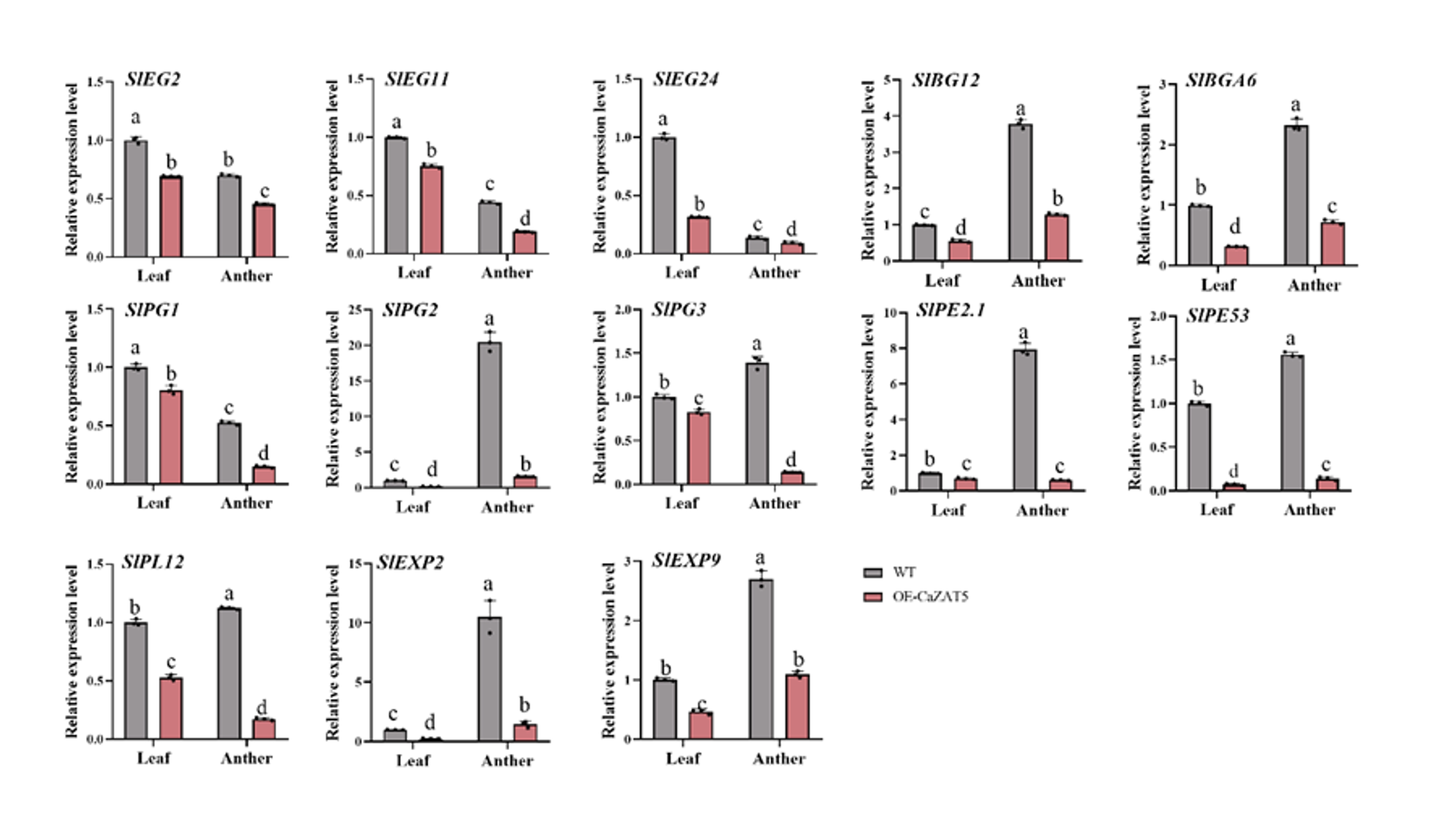

Supplement: S5 Fig — (TIF) [file pgen.1012016.s005.tif]

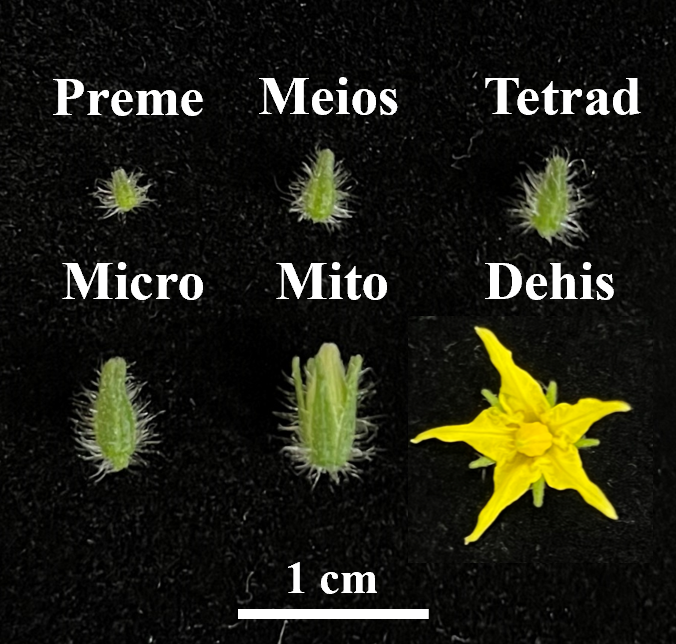

Supplement: S6 Fig — (TIF) [file pgen.1012016.s006.tif]
